# Moms in motion: a group-mediated cognitive-behavioral physical activity intervention

**DOI:** 10.1186/1479-5868-3-23

**Published:** 2006-08-22

**Authors:** Anita G Cramp, Lawrence R Brawley

**Affiliations:** 1 Department of Kinesiology, McMaster University, Hamilton, ON, CA, USA; 2 College of Kinesiology, University of Saskatchewan, Saskatoon, SASK, CA, USA

## Abstract

**Background:**

When examining the prevalence of physical inactivity by gender and age, women over the age of 25 are at an increased risk for sedentary behavior. Childbearing and motherhood have been explored as one possible explanation for this increased risk. Post natal exercise studies to date demonstrate promising physical and psychological outcomes, however few physical activity interventions have been theory-driven and tailored to post natal exercise initiates. The purpose of this study was to compare the effects of a group-mediated cognitive behavioral intervention based upon social-cognitive theory and group dynamics (GMCB) to a standard care postnatal exercise program (SE).

**Method:**

A randomized, two-arm intervention design was used. Fifty-seven post natal women were randomized to one of two conditions: (1) a standard exercise treatment (SE) and (2) a standard exercise treatment plus group-mediated cognitive behavioral intervention (GMCB). Participants in both conditions participated in a four-week intensive phase where participants received standard exercise training. In addition, GMCB participants received self-regulatory behavioral skills training via six group-mediated counseling sessions. Following the intensive phase, participants engaged in a four-week home-based phase of self-structured exercise. Measures of physical activity, barrier efficacy, and proximal outcome expectations were administered and data were analyzed using ANCOVA procedures.

**Results and discussion:**

ANCOVA of change scores for frequency, minutes, and volume of physical activity revealed significant treatment effects over the intensive and home-based phases (p's < 0.01). In addition, ANCOVA of change in mean barrier efficacy and proximal outcome expectations at the conclusion of the intensive phase demonstrated that GMCB participants increased their initial level of barrier efficacy and outcome expectations while SE participants decreased (p < 0.05).

**Conclusion:**

While both exercise programs resulted in improvements to exercise participation, the GMCB intervention produced greater improvement in overall physical activity, barrier efficacy and proximal outcome expectations.

## Background

When examining the prevalence of physical inactivity by gender and age, women over the age of 25 are at an increased risk for sedentary behavior [1-3]. While current research identifies a number of possible barriers to regular physical activity, one particular barrier that occurs for women between the age of 25 and 35 is childbearing and the early years of raising children. These factors have been explored as one possible explanation for the sedentary behavior of young mothers [4]. The post natal period, defined as occurring immediately after birth and lasting up to one year, is filled with new or altered behavioral patterns such as sleepless nights, unregimented feeding patterns, and increased demands of time and physical changes [5]. These demands of parenthood may be barriers that reduce or eliminate the possibility of regular physical activity [6].

Post natal exercise studies demonstrate promising physical and psychological outcomes such as improved cardiovascular fitness, weight loss, increased energy, better mood, and greater confidence in child rearing abilities [7,8]. However, considered within the context of the larger exercise literature, relatively few post natal exercise studies have examined adherence to physical activity as the dependent variable. Furthermore, relatively few of the post natal physical activity interventions have been theory-driven thereby limiting the potential to explain observed effects and understand behavior.

Two recent theory-based post natal interventions are exceptions [9,10]. Miller and colleagues [9] examined the influence of partner support and physical activity self-efficacy relative to the physical activity for women with young children. Participants were recruited through childcare centers and were randomly assigned to one of three conditions: 1) control, 2) receiving print information about overcoming physical activity barriers, and 3) receiving print information plus an invitation to attend discussion groups about the development of strategies for physical activity promotion specifically for mothers. The results of the intervention demonstrated that participants in the third intervention group had a significantly greater number of mothers classified as being physically active compared to control participants. In addition, the investigators demonstrated that the effects of the intervention were partially attributable to changes in the mediating variables of partner support and physical activity self-efficacy.

Furthermore, Fahrenwald and colleagues [10] developed a physical activity intervention based on selected constructs from the Transtheoretical Model (TTM) for women with infants and children. The goal of the intervention was to evaluate progression in stage of behavior change, increase physical activity behavior and facilitate improvements in behavior change constructs of self-efficacy and decisional balance. The results demonstrated that women who received training based on the components of the TTM progressed in the stage of change, improved their physical activity behavior, had higher self-efficacy and a higher ratio of pros to cons for change compared to the control group.

In summary, these studies reflect initial and promising evidence that interventions that focus on changing theory-based behavioral constructs have positive effects on increasing physical activity participation. However, post natal physical activity intervention research is limited and there is a need for more research in this area. Therefore, we considered reviews of theory-based interventions with other unique populations for evidence of successful approaches to enhancing adherence to physical activity.

A review of successful exercise behavior change interventions for older adults highlighted a promising theory-based intervention framed in both social cognitive theory and group dynamics [11]. This intervention model may have application with post natal mothers in that they also face unique challenges and barriers when attempting to adopt a regular physical activity routine. The intervention coupled exercise with group-mediated cognitive-behavioral counseling to foster adherence and improvements in physical function [12]. The theoretically based, group-mediated cognitive behavioral intervention focused upon developing self-efficacy and other social cognitions in regard to encouraging adherence in the initiation and maintenance of physical activity.

In addition to this intervention evidence, physical activity research has consistently identified self-efficacy as playing an important role in exercise participation [13-15]. In fact, self-efficacy is the most frequently identified psychosocial determinant of adherence to physical activity [16,17]. For example, previous research based upon efficacy theory has found a positive relationship between barrier efficacy and exercise behavior such that higher barrier efficacy is associated with a higher frequency of exercise [18]. Furthermore, when individuals are beginning an exercise program, self-efficacy theory [17,18] suggests that outcome expectations play an important role in helping to motivate behavior in addition to the influence of efficacy beliefs [19]. Outcome expectations are defined as the individual's belief that particular courses of action will ultimately produce certain desired outcomes [16].

Using theory and evidence as a guide, we developed and conducted an intervention in a facility-based community environment to increase post natal physical activity behavior. The intervention utilized a theoretically based, group-mediated cognitive behavioral approach [12,20] to motivate post natal mothers to initiate physical activity in a structured setting and to sustain this behavior in their transition to individual home-based exercise. To examine the effects of this approach, a standard exercise program (SE) for post natal mothers was compared to a program that coupled exercise with group-mediated cognitive behavioral counseling (GMCB). It was hypothesized that GMCB participants would demonstrate superior initial and sustained exercise participation, sustained efficacy to overcome barriers, and greater proximal physical outcome expectations compared to participants in the SE condition.

## Methods

### Study sample

Volunteer participants were recruited via a local community newspaper article on post natal fitness. All participants were screened for eligibility upon initial contact and met the following inclusion criteria: (a) still in the post natal period: defined as between 6 and 52 weeks post natal; (b) primarily sedentary: (i.e., less than a daily accumulation of mild to moderate physical activity two or fewer days per week) for the past six months and (c) received consent from their physician to be physically active. In addition, for purposes of participant safety, self-report exclusion criteria at screening included (a) severe heart condition, (b) other medical conditions such as chronic kidney or liver rheumatic disease, cancer, hearing or sight impairment, (c) inability to speak English and (d) currently pregnant. Approval to conduct the study was granted by the University's Research Ethics Office and written consent was obtained from all study participants.

### Intervention setting and conditions

The intensive phase of the intervention was conducted at a community-based fitness facility. The dual city community in which the intervention was conducted has a population of approximately 300,000 blue and white-collar residents. The fitness facility was a large commercial gym and provided its facility free of charge for the purpose of the study with no obligation for participants to enrol during or post investigation. The intervention consisted of two conditions; the SE and the GMCB.

The ***common features of the interventions ***were as follows. Each condition was exposed to a supervised, center-based intensive exercise phase and a participant-managed home-based phase, each phase lasting four weeks. The intensive phase consisted of center-based standard exercise training classes (conducted by a certified fitness instructor) twice a week. Childcare was provided onsite for a nominal fee or participants had the option of bringing their baby into the class. The standard exercise aspect of both treatments was identical in format and included the traditional components of a fitness class: warm up, cardiovascular (aerobic) and strength training, and a cool down/flexibility [21]. Throughout the course of the intensive phase, participants were asked to monitor their physical activity by recording the type, duration and intensity of their daily physical activity in a log book provided by the principal investigator. The purpose of the physical activity log book was to promote self-monitoring only and was not as a measure of physical activity. All participants were encouraged to exercise at home in addition to their structured classes and this encouragement was underscored by the provision of their intensive phase log books. Immediately following the intensive phase, participants handed in their intensive phase log books for interventionist feedback.

The home-based phase began immediately following the end of the intensive phase. During this period, all participants were encouraged to implement their own home-based exercise regime by incorporating exercises learned in class, or essentially any other type of exercise they enjoyed. As in the intensive phase, participants were asked to monitor their physical activity by recording the type, duration and intensity of physical activity they completed in a home-based phase log book given to them at the conclusion of the intensive phase. At the end of the home-based phase, participants were contacted to mail in their log books.

The ***unique aspects of the GMCB intervention ***are summarized below. It should also be noted that this intervention approach has been reported in greater detail for other published studies of both asymptomatic and symptomatic individuals [12,20]. In addition to the common procedures, participants in the GMCB treatment received six, 20-minute group-mediated cognitive behavioral training sessions immediately following the center-based exercise classes over the course of the intensive phase.

The purpose of each 20-minute GMCB session was to have participants focus on developing self-regulatory skills for self-management of physical activity and to overcome post natal specific barriers to self-managed physical activity. To help develop these skills, GMCB sessions included topics such as learning how to self-monitor daily activity, setting goals, and overcoming barriers to physical activity. For example, participants learned the guidelines for effective goal setting, defined barriers, brainstormed a list of barriers and strategies to overcome barriers. In addition, participants created a "back up" physical activity plan they could implement at home in the event that they were unable to engage in their planned physical activity. To reinforce these skills, GMCB participants were encouraged to practice the skills learned in the cognitive-behavioral training session in order to complete home-based physical activity assignments devised through participant-interventionist collaboration. To avoid ongoing group dependency and encourage the practice of self-regulatory behavior while participants were in the program, increasingly greater self-regulation was practiced each week. The GMCB group focused on gradually weaning participants from dependency on the instructor and on the group program toward independent self-regulation of home-based physical activity. During the home-based phase, and consistent with previous studies, one telephone contact at week two of the phase was provided as a final brief opportunity (10 mins) for GMCB participants to review their self-management of activity with staff and to wean participants from further contact or potential dependency.

It is important to emphasize that both treatment conditions received equal total staff contact hours (10 hrs). Contact time was made equivalent by taking into account the delivery of both intervention phases. Staff contact for the SE condition was exercise training only; where two, 75 minute exercise training bouts were provided per week over four weeks (i.e., 150 mins/week × 4 weeks = 600 mins or 10 total hrs). Staff contact time for the GMCB condition was made equivalent to the SE condition by administering (a) exercise training, (b) cognitive behavioral training, and (c) 1 telephone call at the end of the second home-based phase week. Specifically, week one consisted of two 55 minute exercise bouts plus one 20 minute counseling session (130 mins); weeks two and three had two 60 minute exercise bouts plus two, 20 minute counseling sessions per week (160 mins each × 2 = 320 mins); week four had two 60 minute exercise bouts plus one counseling session (140 mins) totalling 590 minutes for the intensive phase Finally, GMCB participants received their single telephone support contact of ten minutes at week two of the home-based phase for a total staff contact of 10 hrs (600 mins).

### Measures

The internal consistency for each scale was verified by calculating Cronbach's alpha. Alpha levels are reported in the description for each measure where applicable. All measures were psychometrically sound according to internal consistency criteria suggested by Tabachnik and Fidell [22].

### Physical Activity (PAR)

The 7-day Physical Activity Recall (PAR) questionnaire was utilized to assess self-reported physical activity [23,24]. The PAR has been successfully validated using objective measures (i.e., oxygen uptake and accelerometer data) [25]. Participants were asked to recall specific activities and estimate the mean frequency and minutes they spent doing each activity in a typical week over the past four weeks. For example, participants indicated in a typical recent week, how many times per week and for how many minutes they engaged in activities such as brisk walking, swimming and resistance training. Each of the measures of frequency per week and minutes per session were summed, and then a mean was calculated for each. To estimate weekly physical activity volume, mean weekly frequency and mean session duration were multiplied. In this investigation, the dependent measure concerned only the moderate to vigorous physical activity, as this was the intensity of activity encouraged for women in each treatment. Thus, further reference to the measure will be to PARmod +.

### Proximal exercise outcome expectations

Proximal outcome expectations were assessed using an outcome expectancy measure [26]. Example items included: weight control, increased fitness, energy and alleviation of bodily pain. Participants rated each outcome in terms of likelihood (i.e., likelihood of the outcome occurring) using a 9-point Likert scale where 1 represented "very unlikely: and 9 represented "very likely". Cronbach's alpha was .87 indicating acceptable internal consistency [25].

### Barrier efficacy

A 17-item modified version of Garcia and King's barrier efficacy scale [27] was administered to all participants as a means of assessing their confidence to address barriers that arise for post natal women in their pursuit to become independent and regular exercisers. Six new items in the modified scale addressed barriers specific to post natal women (i.e., "The amount I am confident that I could be physically active when my child is being fussy is"). Barrier efficacy was measured after the second class to allow for initial mastery experience and prevent an over-estimation of efficacy scores [18]. The efficacy scale ranged from 0 to 100 percent, increasing in 10-point increments, where 0 percent indicated "absolutely not confident" and 100 percent indicated "absolutely confident". Item scores were summed and the sum was divided by the number of items to create an overall scale mean. A Cronbach's alpha of .92 indicated good internal scale consistency [22].

### GMCB intervention checks

In order to check on whether the GMCB intervention factors of group cohesion and interventionist-participant collaboration were perceived, a 5-item group cohesion measure and a 7-item collaboration measure were administered [20]. The cohesion measure was used as an indicator of whether steps to foster group cohesion and group consensus on the goal of adherence were recognized by GMCB participants. An example cohesion item included, "The class members (group) help keep everyone motivated to continue exercising". The interventionist-participant collaboration measure was used to determine if GMCB participants perceived successful collaboration between themselves and their interventionist as this is known to foster participant commitment and adherence [28]. An example collaboration item included "Because of the collaborative discussions post class, I feel I have an independent exercise plan I can implement after the program is done". Possible responses to both measures ranged from 0, "strongly disagree" to 4, "strongly agree" in 1-point increments. Item scores were summed, and the sum was divided by the number of items to create an overall scale mean for each intervention check measure. Internal consistency for the cohesion and collaboration scales were acceptable, α = .67 and α = .83 respectively [22].

### Assessment procedure

Prior to the intensive phase, all participants completed baseline testing. Baseline assessment included the primary test battery of a demographics questionnaire, PAR mod+, outcome expectations, and barriers efficacy. Immediately following the intensive phase, all participants completed the primary test battery again with the exception of the demographics questionnaire. For the GMCB participants, intervention check measures of cohesion and collaboration were assessed at week two and week four of the intensive phase. Finally, at the conclusion of the home-based phase, all participants were contacted to complete the PAR.

### Statistical analysis

Data were analyzed using SPSS (v11.0, 2001). To determine if randomization was effective, all demographic and baseline data were initially examined using ANOVA procedures.

The unit of analyses for each outcome measure was a difference score; calculated as final intervention responses minus baseline values. In the case of PARmod + variables, the change was calculated using eight week post home-based phase PARmod + minus baseline PARmod +. In the case of self-efficacy and proximal outcomes, the change was calculated using four-week post intensive phase values minus baseline values. Each separate analysis included covariates for baseline value for the dependent measure in the respective models. The main outcome variables were analyzed using an ANCOVA procedure. The significance level for all statistical tests was set at p < 0.05. To test for potential covariates in the analyses, correlation analyses were conducted between demographic information and all outcome variables. The means for cohesion and collaboration are reported as intervention checks to ensure that the GMCB intervention was successful at developing these variables.

## Results

### Demographic and baseline data

**Figure 1:**
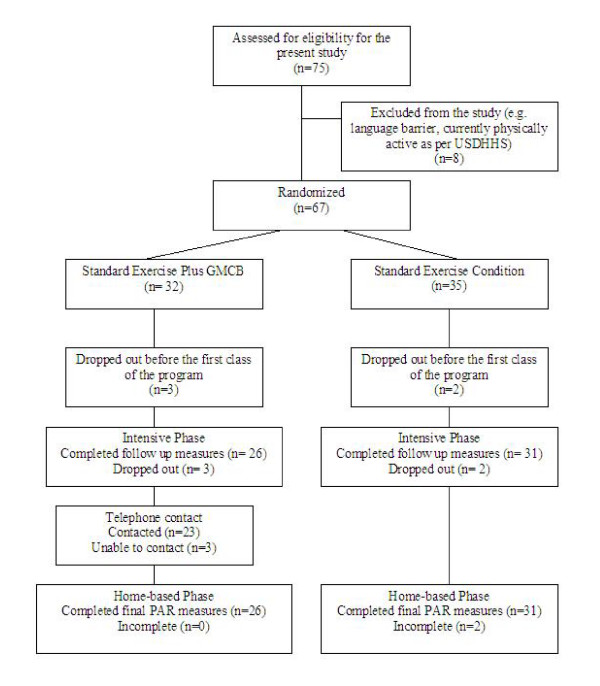
**Flow diagram of participant randomization and follow up data collection**. Figure 1 shows the process of participant inclusion/exclusion and randomization into the intervention treatment arms and subsequent follow up data collection. The figure should be read from top to bottom.

Seventy-five volunteers were recruited and screened for the current study. Of the 75 participants recruited and screened, eight participants were excluded as per the eligibility criteria. Sixty-seven post natal women were randomized into either the Standard Exercise treatment or the Standard Exercise plus Group-Mediated Cognitive Behavioral treatment. A total of 57 participants (SE: n = 31, GMCB: n = 26) completed the intervention. Figure 1 illustrates the randomization process. Randomization was effective for assignment to treatment in that there were no significant differences across demographic variables, baseline PARmod +, outcome expectations and barrier efficacy. The mean sample age was 31.5 years (range: 20 – 46 years) and the mean number of children for the participant sample was 1.6 (range: 1 – 5). Baseline demographic data is displayed in Table 1. Participants completed on average 6 of 8 post natal exercise classes for a 75% attendance rate. A one-way ANOVA revealed no significant between-treatment differences for intensive-phase attendance.

**Table 1:** Participant Demographic Information at Initial Screening by Treatment

Variable	GMCB	SE
Sample	26	31
Mean age^a^	31.23	31.74
Married		
Single	1	0
Married	25	31
Number of children^b^	1.62	1.58
Average month babies were born^c^	6.87	6.73
Breast feeding	11	12
Bottle feeding	15	21

^a^SD = 5.1, Age range = 20 – 46.

^b^Minimum value = 1, maximum value = 5.

^c^All participants gave birth in the year 2003. Month of delivery was coded by number(e.g., 1 = January, 12 = December).

N – Sample Size

GMCB – Group-mediated Cognitive Behavioral Treatment Arm

SE – Stand Exercise Treatment Arm

F- F Statistic

p – Probability Value

### Physical activity

GMCB participants reported a significantly higher change in frequency and volume of physical activity compared to SE participants. An ANCOVA test examining change in mean total frequency of PARmod + revealed a significant treatment effect, F(1,56) = 14.37, p < 0.01 (eta squared = .22). The ANCOVA test for change in mean minutes (duration) per session of PARmod + did not reveal a significant treatment effect. However, an ANCOVA test examining change in mean total volume of PARmod + revealed a significant treatment effect F(1, 52) = 8.36 p < 0.01 (eta squared = .14). Thus, at final assessment, the GMCB group had a significantly overall greater change in PARmod + compared to the SE group. Table 2 shows the mean total frequency, duration and volume of PARmod + for each treatment group at baseline and post home-based phase.

**Table 2:** Raw Means for PARmod + and Social Cognitive Variables

	Baseline		Post Intensive Phase		Post Homs-Based Phase	
	GMCB	SE	GMCB	SE	GMCB	SE
PARmod + Frequency	1.8	1.9	7.7	4.9	6.7	3.65
	(1.7)	(1.8)	(3.2)	(2.2)	(4.08)	(2.4)
PARmod + Duration	38.37	48.78	57.44	57.07	51.82	46.38
	(41.82)	(39.96)	(15.01)	(21.74)	(23.51)	(29.61)
PARmod + Volume	126.34	125.32	444.15	279.67	400.38	222.24
	(152.56)	(131.25)	(218.54)	(147.50)	(288.64)	(177.37)
OE-Physical Proximal^a^	7.36	7.16	7.57	6.56	_	_
	(.99)	(1.24)	(.71)	(1.45)		
Barrier Self-efficacy^b^	6.03	5.47	6.18	5.36	_	_
	(1.20)	(5.36)	(1.49)	(1.28)		

Note. Standard deviations are reported in parentheses under each mean.

^a^Minimum = 1 "very unlikely", Maximum = 9 "very likely".

^b^Minimum = 1 "absolutely not confident", Manimum = 10 "absolutely confident".

GMCB – Group-mediated Cognitive Behavioral Treatment Arm

SE – Standard Exercise Treatment Arm

M – Mean

### Proximal physical outcome expectations

The ANCOVA results for change in mean total proximal physical outcome expectations revealed a significant treatment effect, F(1,56) = 11.32, p < 0.01 (eta squared = .17). GMCB participants had significantly greater changes in proximal outcome expectations than SE participants (estimated change marginal mean: GMCB = .21; SE = -.64). The raw mean scores at baseline and post intensive phase are presented in Table 2.

### Barrier efficacy

The ANCOVA test examining change in mean barrier efficacy revealed a significant treatment effect, F(1, 53) = 10.59, p < 0.05 (eta squared = .17). The SE condition experienced a decline in barrier efficacy from baseline to post intensive phase assessment, whereas the GMCB condition experienced a small increase in barrier efficacy (estimated marginal change mean GMCB = .15; SE = -.14). Table 2 contains the raw mean barrier efficacy scores at baseline and post-intensive phase.

### GMCB intervention checks

Measures of cohesion and collaboration were only taken to verify that perceptions of cohesion and collaboration were created in the GMCB treatment condition. A group-learning environment was created to help GMCB participants learn self-regulatory skills. Thus, measures were obtained to verify GMCB participants' perceptions that these group-learning environment conditions were present [29]. Recall that the measurement scale ranged from 0 (strongly disagree) to 4 (strongly agree). The overall cohesion and collaboration means at the conclusion of the intensive phase indicate that cohesion and collaboration were perceived as conditions that were created and were rated highly by GMCB participants (mean cohesion = 2.96, SD = .29; mean collaboration = 3.19, SD = .36).

## Discussion

The results of the intervention demonstrate that standard exercise training combined with group-mediated cognitive-behavioral counseling (GMCB) provided superior exercise participation effects for frequency, and volume of PARmod+ compared to standard exercise training (SE) alone. While improvements to participants' exercise participation was observed in both exercise programs, the participants in the GMCB intervention had significantly greater improvement in frequency and volume of PARmod+ over the course of the intervention.

An examination of social cognitions indicated significant effects for both proximal outcome expectations for exercise and barrier self-efficacy. In the case of the GMCB participants, values for both outcomes were sustained and even increased slightly. By contrast, for the standard exercise group participants, values for both outcomes declined from those reported at baseline. Reviews of the exercise efficacy literature have also pointed out that early in an exercise program it is not uncommon to observe a decline in participants' initially strong levels of social cognitions [18]. While this decline was observed for the standard exercise group, the GMCB mothers sustained higher values for barrier self-efficacy and proximal outcome expectations. These findings suggest that the mothers who received group-mediated cognitive-behavioral counseling benefited from the intervention in that it encouraged them to maintain their strong social cognitions.

Self-efficacy theory suggests that it is the *combined effect *of both self-efficacy and outcome expectations that lead to more frequent and persistent behavior [17,30]. When the results of both proximal physical outcome expectations and barrier efficacy are considered together with increased exercise participation, it is clear that the GMCB participants increased their expectations for short-term activity outcomes while increasing their efficacy. It is suggested that the incentive derived from achieving proximal outcomes and sustaining barrier efficacy encouraged GMCB participants to increase and then maintain their exercise participation during the follow-up home-based phase where participants self-managed their own activity [17].

This short-term intervention study holds several strengths. The current intervention approach used theoretically based strategies to influence variables identified as facilitators of adherence-related behavior [31,32]. Thus the study adds to the limited number of theoretically driven exercise interventions for post natal mothers. This study also possesses a variety of methodological strengths. Random assignment of participants to treatment groups was used to insure that there were no systematic initial differences between the treatment groups. Study retention was high (85%) and there was no selective effect of retention for either treatment. The use of intervention check measures of cohesion and collaboration affirmed the successful development of a group environment for the learning of self-regulatory skills that was a necessary characteristic of the GMCB intervention [29]. This investigation is one of the few in exercise literature to actually evaluate whether the social context of the experimental treatment intervention was achieved [33]. Furthermore, the results add to those interventions utilizing social-cognitive theory in two ways. First, outcome expectations are rarely examined [19] and second, they have not been previously examined for this unique population.

In as much as this investigation reflects a preliminary study to facilitate adherence in a specific population, it also has limitations. First, study participants were self-selected volunteers, thus results may not be representative of a sample of post natal women who are less motivated to participate in exercise. Second, physical activity participation was self-reported. Whereas PAR has established reliability and validity in comparisons with objective measures [25], it would be useful to include objective physical activity assessment to confirm the reports offered by the PAR measure in future research. Finally, our investigation was of limited tenure and was focused upon initiating and increasing exercise participation through center-based and home-based means. In future, it would be beneficial to determine if physical activity could be sustained during a longer home-based phase. Future research might attempt to conduct a longer investigation that spans the entire post natal period. As this phase is characterized by multiple, unpredictable obstacles that could potentially limit participation in physical activity, it would be important to determine if the positive effects demonstrated in this preliminary intervention could be maintained by mothers as they move beyond the post natal period to continue child-rearing. In addition, it may also be of interest to tailor the intervention differently to accommodate activity KKD levels sufficient to produce health benefits.

## Conclusion

The results of the current investigation demonstrate that a group-mediated cognitive behavioral approach to promoting physical activity initiation among sedentary post natal women produces more favourable effects than standard exercise training alone. This preliminary intervention incorporated the learning, practice, and mastery of strategies to a) overcome barriers, b) foster positive proximal outcome expectations, and c) increase physical activity participation. The post natal period has been identified as a lifecycle time when physical activity is often abandoned [34]. Thus intervening at this stage is crucial for the prevention of long-term physical inactivity. Although more research is needed to examine this issue and investigate the psychosocial determinants of post natal exercise participation, the current study represents a promising initial step in theory-driven, post natal exercise intervention research.

## Competing interests

The author(s) declare that they have no competing interests

## Authors' contributions

AC conceived of the study, recruited participants and carried out all other logistics of running the intervention, performed the statistical analysis and helped to draft the manuscript. LB assisted in the design of the study, performed the statistical analysis and helped draft the manuscript. All authors read and approved the final manuscript.
